# Dynamics of *Theileria equi* Infection in *Rhipicephalus (Boophilus) microplus* during the Parasitic Phase in a Chronically Infected Horse

**DOI:** 10.3390/pathogens11050525

**Published:** 2022-04-29

**Authors:** Maristela Peckle, Huarrisson Santos, Marcus Pires, Claudia Silva, Renata Costa, Gabriela Vitari, Tays Camilo, Nelson Meireles, Patrícia Paulino, Carlos Massard

**Affiliations:** 1 Department of Animal Parasitology, Veterinary Institute, Federal Rural University of Rio de Janeiro—UFRRJ, Seropedica, Rio de Janeiro 23897-970, Brazil; msandes@ufrrj.br (M.P.); claudiabezerra@ufrrj.br (C.S.); renatalins.costa@yahoo.com.br (R.C.); gabriela.vitari@ufrrj.br (G.V.); taysaraujo.medvet@gmail.com (T.C.); meirelesvet@ufrrj.br (N.M.); carlosmassard@ufrrj.br (C.M.); 2 Department of Epidemiology and Public Health, Veterinary Institute, Federal Rural University of Rio de Janeiro—UFRRJ, Seropedica, Rio de Janeiro 23897-970, Brazil; huarrisson@ufrrj.br (H.S.); patgpaulino@ufrrj.br (P.P.)

**Keywords:** trans-stadial transmission, intrastadial transmission, equine theileriosis, ticks, real-time PCR

## Abstract

Experimental studies have demonstrated that *Rhipicephalus* (*Boophilus*) *microplus* transmits *Theileria equi* to horses. However, the degree and dynamics of this protozoan infection in the vector’s organism have not been fully elucidated. Therefore, this study aimed to evaluate the infection rate and parasitic load of *T. equi* in *R. (B.) microplus*, the infection dynamics in this arthropod during experimental infestation in a horse chronically infected with *T. equi*, and to evaluate the trans-stadial and intrastadial transmission competence of *T. equi* by *R. (B.) microplus*. The experimental infestation period of *R. (B.) microplus* on the horse was 33 days, but males were found on the animal up to 60 days post-infestation. After the fifth day post-infestation, ticks and equine blood were collected every two days. Whole ticks from the same developmental stage collected in the same day were pooled. Adult ticks were dissected to extract salivary glands and gut. DNA extraction was performed for all the samples, and they were then submitted to qPCRs for *T. equi* diagnosis. Freshly molted nymphs collected as larvae in the horse and freshly molted males and females collected as nymphs in the horse showed equal to or greater than 75% positivity for *T. equi*, indicating a strong possibility of trans-stadial transmission. The longest permanence of the male ticks on the horse associated with the high positivity rate of this type of sample for *T. equi* indicate that the male may play a role in the intrastadial transmission of *T. equi* to infection-free horses. The salivary glands displayed 77.78% positivity for *T. equi* and presented a higher infection rate at the end of the experimental period (100% from 29 to 33 days post-infection). This study shows that *R. (B.) microplus* has high *T. equi* infection rates and that the infection rate and parasitic load increased over the experimental period. These findings confirm the importance of chronically infected horses with *T. equi* as a source of infection for *R. (B.) microplus.*

## 1. Introduction

Equine piroplasmosis (EP) is a disease that affects horses and presents, in the acute phase of the disease, clinical features such as fever, anemia, jaundice, hemoglobinuria, and weakness [1,2]. The etiological agents responsible for this disease around the world are the hematozoa *Theileria equi* (Laveran, 1901) Mehlhorn and Schein, 1998, *Theileria haneyi* Knowles & Kappmeyer, 2018 [3], and *Babesia caballi* (Nuttall and Strickland, 1912). In addition, horses can become chronically infected for life and function as reservoirs of EP agents for tick vectors [4].

Under natural conditions, these pathogens are transmitted by over 20 tick species of several genera, including *Dermacentor*, *Rhipicephalus*, *Hyalomma*, *Haemaphysalis*, and *Ixodes* ticks [5,6]. In Brazil, the tick species indicated as the primary vectors in the transmission of *T. equi* and *B. caballi* are *Rhipicephalus (Boophilus) microplus* [7] and *Dermacentor nitens* [8], respectively. *Rhipicephalus (B.) microplus* is a one-host tick of cattle, and horses, caprines, and dogs are secondary hosts [9,10]. Consequently, parasitism in horses depends on the presence of cattle, the primary hosts, in the conditions of intercropped pastures in which both species are raised together [11,12].

Under experimental conditions, *T. equi* sporoblasts and sporozoites are identified in the salivary glands of *R. (B.) microplus* [13]. Subsequently, the trans-stadial transmission of *T. equi* by *R. (B.) microplus* was confirmed through the infestation of hemoparasite-free horses by adult ticks infected at the nymph stage during feeding in foals with high parasitemia [7]. Using the nested PCR technique, Battsetseg et al. [14] detected *T. equi* and *B. caballi* DNA in naturally infected *R. (B.) microplus* eggs from Brazil, suggesting that both protozoa are transmitted transovarially and trans-stadially by the tick vector. Subsequently, Ueti et al. [4,15] confirmed the participation of *R. (B.) microplus* as a biological vector of *T. equi* in the trans-stadial and intrastadial modalities. They also demonstrated that chronically infected horses with low parasitemia are competent reservoirs for tick vectors in the transmission of *T. equi*. Although many experimental studies have demonstrated that *R. (B.) microplus* is capable of being infected and transmitting *T. equi* to equines, its role in the epidemiological chain of transmission in Brazil is still unknown [16], especially since it is a one-host tick and horses are its alternative host [17].

It is believed that, under natural conditions, most *T. equi* infections in horses are caused by ticks feeding on chronically infected animals. Once infected by *T. equi*, the microorganism remains in low parasitemia throughout the animal’s life [4,18]. However, little is known regarding the infection rate of tick vectors, the dynamics of infection within the ixodid, and the parasitic load, especially in *R. (B.) microplus*. Therefore, the objectives of this study were to demonstrate the dynamics of *T. equi* infection in *R. (B.) microplus* fed on a chronically infected horse through the infection rate and parasitic load in pooled tick samples and pooled ixodid organs at different stages of their life cycle during the experimental infestation, to evaluate the trans-stadial and intrastadial transmission competence of *T. equi* by *R. (B.) microplus*, and to verify if a chronically infected horse can act as a reservoir of *T. equi* to tick vectors.

## 2. Results

### 2.1. Detection Limit and Efficiency of qPCR in Whole Ticks and Tick Organ Samples

**Figure 1:**
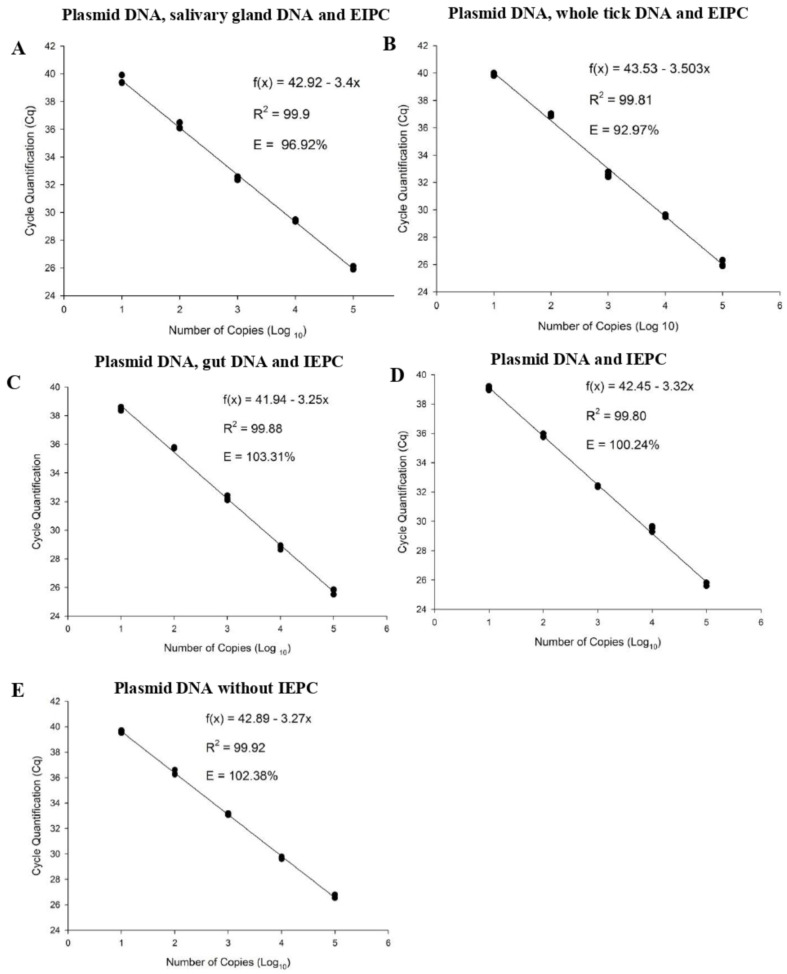
The standard curve (SC) was obtained from serial decimal dilutions (10^5^ copies to 10 copies) of plasmid DNA containing the 18S rDNA fragment of *Theileira equi*. (**A**)—Standard curve (SC) constructed in the presence of salivary gland DNA from infection-free *Rhipicephalus (Boophilus) microplus* and the exogenous internal positive control (EIPC). (**B**) = SC in the presence of the whole DNA of infection-free *R. (B.) microplus* tick and the EIPC. (**C**) = SC in the presence of gut DNA obtained from the infection-free *R. (B.) microplus* and the EIPC. (**D**) = SC only with *T. equi* plasmid DNA and the EIPC. (**E**) = SC with *T. equi* plasmid DNA in the absence of the EIPC. EIPC: preoptimized internal positive control, which can be spiked into samples to distinguish true target negatives from PCR inhibition (TaqMan^®^ Exogenous Internal Positive Control VIC™ Probe).

The qPCR applied in the present study presented a detection limit of ten copies of plasmid DNA under all the conditions tested (Figure 1).

The efficiency of the qPCR reaction was 96.92% when the curve was constructed in the presence of salivary gland DNA from infection-free ticks, 92.97% when constructed in the presence of whole tick DNA, 103.31% in the presence of gut DNA, 100.24% when the curve was constructed with only plasmid DNA and the internal control, and, finally, 102.38% when the curve was constructed with plasmid DNA only. In none of the curves did the presence of DNA from tick tissues cause changes in the Cq of the internal control, and this fact was observed at all the dilutions tested. Thus, the differences in the qPCR efficiencies observed in the SCs constructed in the presence of gut and whole tick DNA can be attributed mainly to the nature of the sample itself. In all the curves, the correlation coefficient of the three replicates of the five dilutions tested was 99%, which means that all the SC assays showed high reproducibility at all the tested dilution points.

### 2.2. Dynamics of Theileria equi Infection in Whole Rhipicephalus (Boophilus) microplus Tick Samples

Regarding the evolutionary stage of *R. (B.) microplus*, engorged larvae were observed on the animal at 5 to 13 days post-infestation (DPI). Nymphs were found on the animal between 5 and 21 DPI. Males were observed between 17 and 33 DPI and females between 13 and 33 DPI. During an inspection of the horse, males of *R. (B.) microplus* were still observed over the animal approximately 60 days after the beginning of the parasitic phase.

Among the whole tick pooled samples, 11 larvae pools, 20 nymph pools, 47 female pools, and 27 male pools were tested by qPCR for *T. equi*, totaling 105 pooled samples. It was observed that 90.9% (*n* = 10/11) of the larvae, 95% (*n* = 19/20) of the nymphs, 97.9% (*n* = 46/47) of the female ticks, and 92.6% of males (*n* = 25/27) were considered *T. equi*-positive by qPCR. Of these samplings, 15 were freshly molted ticks.

Among the nymphs, four samples were freshly molted nymphs (*n* = 4/20). The trans-stadial infection rate from larvae to nymph was 75% (*n* = 3/4), considering they were pooled samples. Regarding the male pooled samples, four were freshly molted male ticks (*n* = 4/27), and the trans-stadial transmission rate from nymphs to males was also 75% (*n* = 3/4). Regarding the 47 pooled samples of female ticks, seven were freshly molted, and just one was considered negative for *T. equi*. Therefore, the trans-stadial infection rate of *T. equi* in ticks collected as nymphs and analyzed as female pooled samples was 85.71% (*n* = 6/7).

The mean value of the parasitic load quantified by qPCR for the pooled samples of whole ticks was 10^5.778^. A significant difference was observed between the mean values of the parasitic load of the whole tick pooled samples as a function of the five periods of the parasitic life cycle of the evaluated ticks in the present study (*p* < 0.0001). Thus, it is possible to demonstrate that, regardless of the ticks’ stage of development, the average parasitic load of these specimens tends to increase with the advancement in the tick cycle on the horse (Table 1).

The average *T. equi* load in the whole ticks pooled samples increased as a function of the developmental stage and according to the period of the tick’s cycle on the horse. This increase was significant (*p* < 0.05) when the nymph stage was analyzed separately in the periods evaluated. It was possible to observe that there was an increase in the average parasitic load between the first (5 to 9 DPI) and the third (17 to 21 DPI) experimental period in the nymph pooled samples (C1 = 10^2.555^; C3 = 10^5.400^). However, this finding was not observed when adult pooled samples were analyzed. No significant differences were observed between the average parasitic load of the pooled samples of males and females in the three experimental periods analyzed (Table 2).

**Figure 2:**
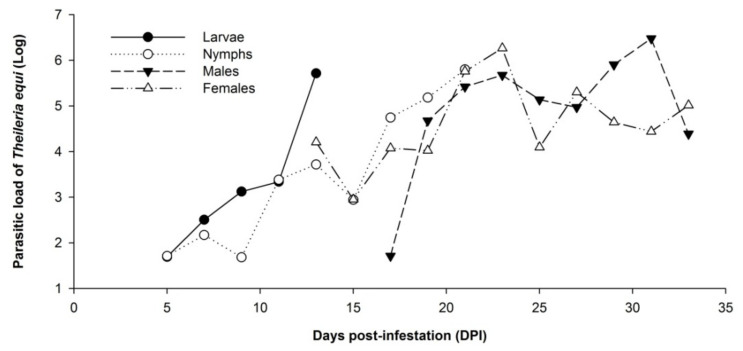
Parasitic load of *Theileria equi* in whole *R. (B.) microplus* pooled samples collected every two days post-infestation from day 5 to 33 of the experimental period.

In general, the parasitic load of *T. equi* in whole tick pooled samples measured every two days increased as a function of the developmental stage and according to the period of the tick’s cycle on the horse (Figure 2).

### 2.3. Dynamics of Infection with Theileria equi in Salivary Glands and Guts of Rhipicephalus (Boophilus) microplus

Forty-five samples from salivary gland pools of adults of *R. (B.) microplus* were analyzed. Of these, 14 pooled samples were from males and 31 from females. Among the male pooled samples, 64.29% (*n* = 9/14) were considered positive by qPCR. In the female samples, 83.87% (*n* = 26/31) were positive for *T. equi*. There was no significant difference in the frequency of positivity in the samples of males and females analyzed.

Of the *R. (B.) microplus* gut pools sampled, 42 samples were analyzed. Among them, 12 were from the male gut and 30 samples from the female gut. Fifty percent (*n* = 6/12) of the male gut pooled samples were considered positive by qPCR. Of the female gut pooled samples, 83.30% (*n* = 25/30) were positive for *T. equi*. The frequency of positivity of the gut samples from females was significantly higher (*p* < 0.05) than the frequency observed in males. The total number of gut samples from these ticks was slightly lower than the number of salivary gland samples due to the fragility of this tissue and the difficulty in removing and separating this organ during arthropod dissection.

When the positivity rate for *T. equi* in the pooled salivary gland samples of adult ticks of *R. (B.) microplus* was compared to the salivary glands of young adults, the frequency was 88.2% (*n* = 30/34), a percentage significantly higher than the 45.5% (*n* = 5/11) observed in freshly molted adult ticks that underwent ecdysis in a Bio-Oxygen Demand chamber (BOD). The frequency of positivity of gut pooled samples from adult ticks was 77.14% (*n* = 27/35), a value noticeably higher than that observed in freshly molted adult ticks (57.14%; *n* = 4/7) but with no significant difference (*p* > 0.05).

A significant difference was observed regarding the frequency of positivity in *T. equi* in the pooled salivary gland samples from adults in each established experimental period (17–21, 23–27, and 29–33). Between 17 and 21 DPI, 47.03% (*n* = 8/17) were considered positive for *T. equi*, differing from the positivity in the second period (93.95%; *n* = 15/16) and the third (100%; *n* = 12/12) experimental period.

Between 17 and 21 DPI, 53.85% (*n* = 7/13) of the pooled adult gut samples were positive for *T. equi*. In the second period, positivity was 76.47% (*n* = 13/17), and, in the third, it was 91.67% (*n* = 11/12). Despite the clear increase in the frequency in gut samples from adult ticks with the progression of the experimental period, it was not possible to observe a statistical difference between these data.

**Figure 3:**
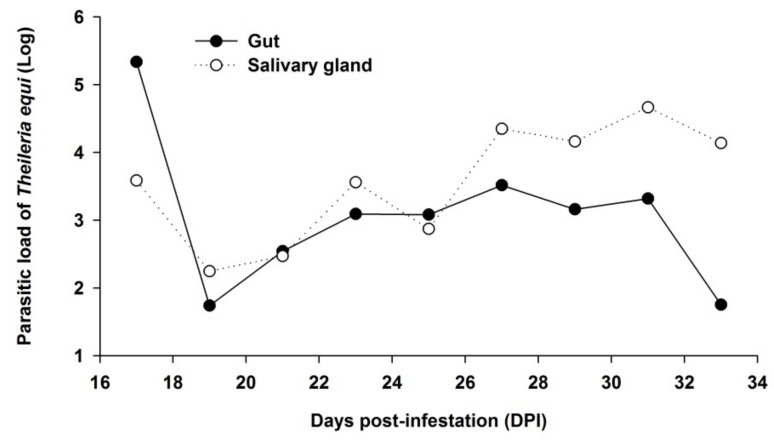
Parasitic load of *T. equi* in the pooled salivary gland and gut samples *of R. (B.) microplus* collected every two days post-infestation from day 17 to 33 of the experimental period.

Regarding the mean values of the parasitic load of *T. equi* in the pooled salivary gland samples and gut samples from positive adult ticks in the three experimental periods tested (17–21, 23–27, and 29–33 DPI), there were no statistical differences (gut: *p-value* = 0.57; salivary gland: *p-value* = 0.08). However, the mean value of the parasitic load in the salivary gland and gut samples from adult ticks tended to increase with DPI. At 31 DPIs, the highest parasitic loads in the pooled salivary gland samples (10^4.667^) and gut samples (10^4.581^) were observed. Conversely, the lowest mean parasitic load was observed at 19 DPI in both salivary gland samples (10^2.248^) and gut samples (10^2.051^) (Figure 3).

Furthermore, the dynamics of the *T. equi* parasitic load have an upward trend in the salivary gland pooled samples, and the opposite was observed regarding the gut pooled samples as the experimental period went by (Figure 3).

### 2.4. Stability of Parasitic Load of Theileria equi during the Experimental Period in the Equine Chronically Infected with Rhipicephalus (Boophilus) microplus

Although an increase in the *T. equi* parasitic load was observed in the whole pooled *R. (B.) microplus* ticks and tick organs, this trend was not confirmed when evaluating the average parasitic load obtained from the analysis of equine blood in the different periods of the experimental phase (*p* = 0.29). The mean value of the parasitic load/µL of the blood of the infected horse remained stable throughout the evaluation period of the parasite life of the tick on the horse ($\bar{x}$ = 10^4.567^; SD = 10^4.320^). In the first period, between 5 and 9 DPI, the average load was 10^4.693^, followed by the evaluation between 11 and 15 DPI, when the average load was 10^4.908^. Between 17 and 21 DPI, the average was 10^4.683^. In the fourth period, between 23 and 27 DPI, the average was 10^4.577^, and, in the final experimental period, the average was 10^4.655^. Thus, a slight fluctuation in the parasitic load of *T. equi* in the chronically infected horse’s blood was observed despite the infestation by *R. (B.) microplus*. This slight oscillation shows that equine infection was stable throughout the infestation period. 

## 3. Discussion

### 3.1. Remarks on Rhipicephalus (Boophilus) microplus Life Cycle in Equine

*Rhipicephalus (Boophilus) microplus* is one of the leading health problems for livestock in many countries, including Brazil. The main hosts are cattle, but it can occasionally parasitize sheep, deer, horses, buffaloes, pigs, goats, dogs [19,20,21,22], and even man [23]. Its pathogenic effects can be classified as direct by the action of hematophagy in all the stages of tick development and indirect by the transmission of pathogens, such as *Babesia* spp. and *Anaplasma* spp.

The presence of this tick species on horses from different regions of Brazil has been reported by several authors [12,24,25]. The average period of the parasitic phase of *R. (B.) microplus* on the horse in this study was 33 days, corroborating the studies performed by Bittencourt [25] and Franque et al. [22], who observed 28 to 38 days of parasitic phase. In its preferred host, cattle, the onset of the shedding of engorged females occurs between 19 and 21 days [26]; however, males could stay on the host for up to 60 days, which was also observed in this study.

### 3.2. Transmission of Theileria equi by Ticks in Brazil

Ticks of the genera *Dermacentor*, *Rhipicephalus*, *Hyalomma*, *Haemaphysalis*, and *Ixodes* have been described as transmitters of *T. equi* around the world [6]. In Brazil, three species of ticks are observed to parasitize horses: *Dermacentor nitens*, *Amblyomma sculptum*, and *R. (B.) microplus* [12,27]. Of them, only *R. (B.) microplus* is indicated as a possible biological vector of *T. equi* in horses [4,7,15,28]. Although *A. sculptum* (*A. cajennense* complex) is a possible vector based on epidemiological data [11,16,29,30], the infection of *A. sculptum* nymphs by *T. equi* is not observed under experimental conditions [31].

### 3.3. Chronically Infected Equines as Reservoirs of Theileria equi

The use of horses chronically infected with *T. equi* mimics what occurs under field conditions, where horses, once infected, maintain this theileriid at a low parasitemia for life and function as an efficient source of infection for vector ticks. Some studies have shown that, once a threshold parasitic load in the blood of the vertebrate host is reached, either in the acute phase of infection (10^8.8^ parasites/mL of blood on average) or the chronic phase (10^5.5^ parasites/mL of blood in average), a further increase in the number of pathogens in the blood has little or no effect on the percentage of infected ticks [4,32,33]. Ueti et al. [4,15] reported that parasitemia during tick feeding affects the efficiency of the initial infection. However, infected ticks have similar parasitic load levels in the salivary glands regardless of equine parasitemia during feeding and transmit the pathogen with the same efficiency. Therefore, an experimental model using a chronically infected horse is equally efficient in studies of the transmission of this hemoparasite. This efficiency was also demonstrated in the present study, where the positivity for *T. equi* in the whole tick samples was above 90%, and, in the 45 samples of salivary glands of adult ticks, it was 77.78%.

### 3.4. Trans-Stadial Transmission of Theileria equi by Rhipicephalus (Boophilus) microplus

Transmission of *T. equi* by adults of *R. (B.) microplus* fed as nymphs on a chronically infected horse with low parasitemia was demonstrated by Ueti et al. [4,15]. The trans-stadial transmission of *T. equi* through *R. (B.) microplus* nymphs has also been demonstrated [28]. In the present study, it was possible to observe through freshly molted ticks in BOD (freshly molted nymphs, males, and females) the trans-stadial transmission competence of *T. equi* by *R. (B.) microplus* with rates of 75%, in the case of freshly molted nymphs and male ticks, and 85.71%, in the case of freshly molted female ticks. In freshly molted adult ticks, it is suggested that the horse’s blood has already been digested in the intestine as they were taken out as nymphs and the ecdysis shift occurred in BOD. Another aspect that indicates the trans-stadial transmission is the positivity of 45.5% (*n* = 5/11) of the salivary gland samples from freshly molted adults taken from the equine as nymphs. Remarkably, trans-stadial transmission varies with the developmental stage of the tick, and the success of this transmission modality is most significant in the nymph-to-adult molting stage.

### 3.5. Intrastadial Transmission: Is the Male Guilty?

Intrastadial transmission can be considered an essential aspect in maintaining the transmission cycle of *T. equi* to equines in areas where only the species *R. (B.) microplus* is recognized as a biological vector, as happens in Brazil. The ability of male ticks to become infected at a high percentage may explain the high endemic rate of *T. equi* in Brazil since males of this tick genus can feed on more than one host [34]. This fact is reinforced by a study conducted by Ueti et al. [15] in which they observed that only two males with loads of 10^4.2^ parasites per salivary gland are required for the transmission of *T. equi* to an infection-free horse. Ueti et al. [15] highlight the importance of the male due to the behavior of interrupting feeding and greater locomotion between hosts in search of females. Furthermore, the intrastadial transmission of *T. equi* by males of *R. (B.) microplus* was investigated and confirmed, with males being indicated as a vital control target to prevent theileriosis in horses [15].

In addition, the greater permanence of males on the horse, observed in this work at approximately 60 days, associated with the high positivity of this type of sample, indicates that the male may have a significant role in the epidemiological chain of transmission of *T. equi* in Brazil.

### 3.6. Dynamics of Infection of Theileria equi in Rhipicephalus (Boophilus) microplus

The piroplasmid development in the tick vector depends on the balance between the establishment of the tick’s defense against the parasite and the ability of the parasite to escape the tick’s immune response [35]. Studies demonstrate the existence of proteases in the intestine of ticks, responsible for controlling the number of parasites that leave or invade this tissue [35,36], a fact that can balance the protozoan infection in the organ and limit the number of new parasites acquired by the tick while it continues to feed.

In adult ticks, there was a tendency to increase the frequency of positivity during the infestation period in whole ticks (males and females) and in the salivary gland and gut. This fact seems to be related to the time that ticks spent feeding on the chronically infected horse, suggesting that, the longer this time, the greater the frequency of positivity of *T. equi* in ticks and their tissues [37]. In gut samples, 53.85% were positive for *T. equi* at 17 to 21 DPI, rising to 91.67% at 29 to 33 DPI. A study developed by Ueti et al. [37] demonstrated that nymphs feeding for 2 to 3 days on an infected animal showed no detectable *T. equi* infection in gut samples by the direct immunofluorescence technique. However, in nymphs feeding for 7 to 8 days, it was possible to detect the agent in 66 to 100% of the gut samples. Furthermore, Mehlhorn and Schein [38] observed that, after ingestion of gamonts by the tick, the forms remain the same for two days in the gut contents, and zygotes are only visualized between 4 and 7 days after the ingestion of the gamonts.

The present study demonstrates that the load of *T. equi* in the samples of whole ticks was statistically higher as the experimental period progressed (Table 1; Figure 2). When the nymphs, females, and males were analyzed separately, the parasitic load was statistically higher in the third period (17 to 21 DPI) in the case of nymphs, showing an increase in the parasitic load from 17 DPI (Table 2). This trend of increasing load with time was observed for both males and females, without significant difference (Table 2; Figure 2). The same trend of increase was observed in the salivary gland pooled samples, demonstrating that the tick parasitic load tends to increase with the advancement of the experimental period (Table 3; Figure 3).

Oppositely, in the gut pooled samples, there was a tendency to reduce the parasitic load as the experimental period progresses (Figure 3). These infection dynamics in *R. (B.) microplus* can be correlated with the life cycle of *T. equi* inside the tick as gametogony occurs in the gut and results in ookinetes that leave the gut to reach the salivary glands, where the sporogony process occurs, giving rise to multinucleated sporonts producing numerous sporozoites [38].

Interesting results regarding the parasitic load of *T. equi* and infection rate in the *R. (B.) microplus* samples were observed in this study, although the samples analyzed were composed of ticks pooled in the same sample. The pooled sample approach was used as a recourse to increase the DNA quantity per sample as the ticks collected were smaller than the standard found for this species and in a reduced number. Horses are not the favorite host of *R. (B.) microplus*, and, in that case, ticks take more time to complete their life cycle in equines; the recuperation load of tick specimens is low and smaller than ticks recovered from bovines [22]. The percentage of positivity observed in the present study for *T. equi* in whole tick samples was always higher than 90% in all the stages of development of *R. (B.) microplus*. Although it was not possible to determine the rate of individual tick infection at each stage of tick development, it was evident that there is an upward trend in parasitic load throughout the infestation period demonstrated in the salivary gland pools and whole adult tick pool samples. Additionally, the pooled sample approach was applied to reduce the standard deviation between the technical replicates in qPCR as a small quantity of DNA presents poor repeatability in this technique.

It is clear that *R. (B.) microplus* is capable of becoming infected with *T. equi,* and there are strong indications of the influence of time on the positivity of ticks and organs of *R. (B.) microplus* feeding on a chronically infected horse, as well as the increase in the parasitic load over the experimental period.

## 4. Materials and Methods

### 4.1. Hemoparasite-Free Tick Colonies

A colony of *R. (B.) microplus* was obtained from ticks fed on cattle free of hemoparasites by drug sterilization. The engorged females were kept in a BOD-acclimatized chamber at a temperature of 27 °C (±1 °C) and relative humidity above 80% for laying [25,39,40,41,42]. Only the postures of the teleogens from the first 24 h were separated [7], weighed, and transferred to adapted sterile disposable syringes [42], which were kept under the same BOD conditions described above for incubation and hatching of larvae.

### 4.2. Tick Acquisition by Feeding on a Chronically Infected Horse

**Figure 4:**
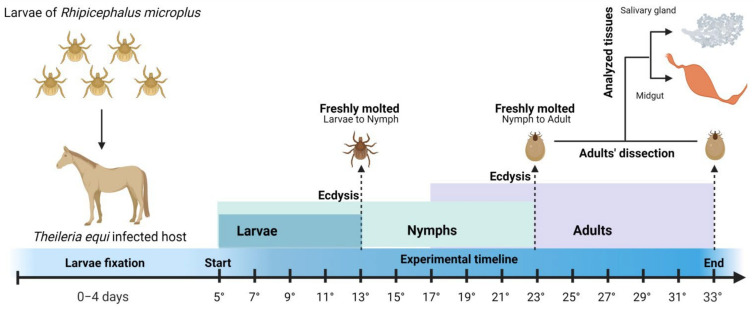
Experimental methodology that demonstrates the sample type and the collection scheme of whole ticks and tick organs to assess the infection rate, parasitic load, and trans-stadial transmission of *Theileria equi* to *Rhipicephalus (Boophilus) microplus* feeding on a chronically infected horse.

A horse chronically infected with *T. equi* was infested with 4 g of hatched *R. (B.) microplus* larvae after 15 days of fasting in BOD. The experimental tests on the horse were carried out over 33 days, with tick specimens being collected from the fifth day post-attachment (5° DPA; Figure 4). From this date, approximately 60 tick specimens were collected every two days. The infestation on the horse was monitored for up to 60 days after the experimental phase.

### 4.3. Tick and Blood Collection

The tick specimens were collected from the horse and arranged in “pools” by evolutionary stage, sex, and collection day. Larvae, nymphs, adults, and freshly molted adult ticks were stored in pools of 20, 5, 3–5, and 2–3 specimens, respectively. Engorged larvae found between 5 and 13 DPI were removed and kept in an acclimatized chamber at 27 °C (±1 °C) and relative humidity above 80% until they completed the ecdysis for nymphs in BOD. Nymphs, fed from the larval stage until their engorgement (7–27 DPI), were carefully removed and kept in an air-conditioned chamber at 27 °C (±1 °C) and relative humidity above 80% until they completed the ecdysis to newly emerged adults in BOD. These samples were used to evaluate the trans-stadial competence of *T. equi* transmission by *R. (B.) microplus*.

A portion of the fed tick specimens divided into “pools” by evolutionary stage, sex, and collection day was stored at −80 °C for further DNA extraction and molecular detection of *T. equi*. Another portion was dissected to obtain salivary glands and intestines according to the methodology described by Edwards et al. [43]. Pooled salivary gland and gut samples were stored in sterile PBS at −80 °C. A scheme of this experiment with *R. (B.) microplus* feeding on a horse chronically infected with *T. equi* is shown in Figure 4.

The level of parasitemia of the chronically infected horse was evaluated by analyzing blood samples collected every two days during the infestation into vacuum tubes containing ethylene diamine tetra-acetic acid (EDTA) through the jugular vein puncture. The collection of blood from the horse was performed simultaneously with the collection of ticks.

### 4.4. DNA Extraction

In all samples from pools of whole ticks and tick tissues, DNA extraction was performed using a commercial kit (DNeasy Blood and Tissue kit, Qiagen^®^, Hilden, Germany) according to the manufacturer’s recommendations. In addition, DNA extraction from the blood of the horse chronically infected by *T. equi* was performed using 300 µL of whole blood, using the Wizard Genomic DNA Purification kit (Promega^®^, Madison, WI, USA) according to the manufacturer’s recommendations.

All equine blood DNA samples were adjusted to a concentration of 100 ng/µL, and DNA samples obtained from ticks and tick tissues were adjusted to 30 ng/µL.

### 4.5. Real-Time PCR Reaction (qPCR)

Molecular detection and determination of the load of *T. equi* were performed using a hydrolysis probe (TaqMan^®^, Applied Biosystems®, Foster City, CA, USA) and qPCR for amplification of an 81 bp fragment of the 18S rDNA fragment [44]. Reactions were performed in duplicate, with a final volume of 12 μL containing: 1X of TaqMan^®^ Universal PCR Master Mix 2x (Applied Biosystems^®^, Foster City, CA, USA), 450 nM of each primer, 250 nM of the probe, and approximately 3µL of the DNA. Thermocycling conditions were 50 °C for 2 min, 95 °C for 10 min, and 45 cycles at 95 °C for 20 s, followed by 55 °C for 1 min [44]. Samples with a quantification cycle (Cq) of ≤40 cycles were considered positive.

### 4.6. Determination of Theileria equi Levels in the Peripheral Blood, Ticks, and Tick Tissues Using Real-Time PCR

The analytical sensitivity of the qPCR assay was determined by evaluating serial decimal dilutions of the 18S rDNA gene cloned into plasmid pGEM-T^®^ Easy Vector System (Promega, Madison, WI, USA). Plasmid DNA concentration was measured in a Qubit^®^ fluorometer (Thermo Fisher Scientific, Waltham, MA, USA), and its purity was evaluated in a Nanodrop^®^ ND-2000 spectrophotometer (Thermo Fisher Scientific, Wilmington, DE, USA). The concentration of plasmid DNA served as a basis for calculating the number of copies of the gene and the average load of parasites in the different DNA samples analyzed. All samples were analyzed in duplicate, on different plates, and the data are presented as the logarithm of the number of copies of the *T. equi* 18S rDNA fragment per microliter of DNA. All calculations were made based on formulas determined by the SCs of relative quantification of each type of sample presented in this study.

In order to determine the precision of the replicas in the qPCR, an SC composed of five points was made, plotting the number of copies of the plasmid against the values of Cq of each replica. From this, the correlation coefficient was calculated. The efficiency of each reaction was determined considering the slope of the SC using the following formula: [Efficiency = 10(−1/slope) − 1].

Efficiency and analytical sensitivity of the qPCR reaction were evaluated with and without the addition of 30ng of whole tick DNA, 30ng of pooled salivary gland DNA, and 30ng of pooled gut DNA in a reaction containing TaqMan^®^ Exogenous Internal Positive Control VIC™ Probe (EIPC). The internal control was added to evaluate the presence of inhibitors in the qPCR reactions, the impact of using the control on the detection of *T. equi* DNA, and the efficiency of the reaction. The reactions were also analyzed in the absence of EIPC. A comparison between the assays was performed by statistical analysis. All DNA samples of ticks and organs used to evaluate the efficiency of the reaction and the analytical sensitivity of the technique were acquired from a colony of ticks free of hemoparasites.

### 4.7. Experimental Design and Statistical Analysis

Throughout the whole experimental period, DNA samples from whole ticks and individual organs of these arthropods were evaluated for the frequency of positivity and the mean parasitic load of *T. equi*. The analyses were performed according to the DPI, with five experimental periods highlighted (5–9, 11–15, 17–21, 23–27, and 29–33 DPI). The values of infection rates and mean parasitic load of *T. equi* in the whole ticks were compared as a function of these selected periods. Regarding tick organs, the same comparative analysis was performed but in only three of these experimental periods (17–21, 23–27, and 29–33 DPI). In both types of samples of DNA pools (whole ticks and organs), the comparison was performed for different stages of development and specimen sex.

The Lilliefors test was used at 5% significance to assess the normality of the data. When the data presented a normal distribution (parametric), ANOVA was performed using the F test followed by the Tukey post-test at 5% significance. When the data distribution was not normal (non-parametric), the Kruskal–Wallis test was used, followed by the Dunn test at 5% significance.

The frequency of positivity of the total pools of the different tissues evaluated (salivary gland and gut) was calculated according to the established experimental period (17–21; 23–27, and 29–33 DPI), depending on the sex of the tick (male or female) and its developmental stage (freshly molted adult or adult). These frequencies were compared using the chi-square or Fisher’s Exact test at 5% significance level.

When the number of observations was ≤5, statistical tests were not performed for frequency analysis or analysis of the average parasitic load variance.

Furthermore, to evaluate the efficiency of using the internal control in the qPCR reactions, the results obtained from the mean values of Cq with and without the EIPC were compared using the Kruskal–Wallis test at 5% significance.

All analyses were performed using the BioEstat 5.0 software [45].

## 5. Conclusions

When feeding on a chronically infected horse, *Rhipicephalus (Boophilus) microplus* can become infected with *Theileria equi*, and their parasitic load rises as the experimental period progresses, confirming the role of the chronically infected horse as a reservoir and source of infection for tick vectors.

Freshly molted nymphs and adults of *R. (B.) microplus,* analyzed as pooled samples, and salivary gland samples from freshly molted adults taken from the equine as nymphs were positive for *T. equi,* which is a significant indication of trans-stadial transmission.

The great permanence of *R. (B.) microplus* males on the horse, associated with a high positivity in the whole tick male samples and in the salivary gland samples of males, indicates that males of *R. (B.) microplus* may play a role in the intrastadial transmission of *T. equi* to an infection-free equine.

## Figures and Tables

**Table 1:** Parasitic load obtained from molecular detection by qPCR in pooled DNA samples of whole *Rhipicephalus (Boophilus) microplus* ticks, irrespective of the arthropod’s sex and stage of development.

DPI	*n*	$\bar{x}$	Md	SD	SE	Min	Max
5–9	10	10^2.699^ _c_	10^2.412^	10^2.825^	10^2.326^	11.7	10^3.309^
11–15	12	10^4.750^ _bc_	10^3.284^	10^5.173^	10^4.634^	45.5	10^5.715^
17–21	21	10^5.651^ _ab_	10^4.640^	10^6.041^	10^5.382^	51.6	10^6.636^
23–27	33	10^6.204^ _a_	10^5.603^	10^6.454^	10^5.695^	13.4	10^7.117^
29–33	13	10^5.953^ _a_	10^4.686^	10^6.220^	10^5.664^	10^3.418^	10^6.742^

DPI: days post-infestation; *n*: number of observations; $\bar{x}$: arithmetic mean; Md: median; SD: standard deviation; SE: standard error; Min: minimum; Max: maximum. Values followed by the same letter in the same column do not differ significantly by the Kruskal–Wallis test at 5% significance (*p* < 0.05).

**Table 2:** Mean parasitic load obtained from molecular detection by qPCR of *Theileria equi* in pools of whole nymphs, male, and female ticks of *Rhipicephalus (Boophilus) microplus* during the experimental infestation period.

DPI	*n*	$\bar{x}$	Md	SD	SE	Min	Max
**Nymphs**							
5–9	4	10^2.555^_a_	10^2.032^	10^2.758^	10^2.457^	11.7	10^3.083^
11–15	8	10^4.235^_ab_	10^3.184^	10^4.590^	10^4.139^	45.5	10^5.053^
17–21	4	10^5.400^_b_	10^5.201^	10^5.416^	10^5.115^	10^4.746^	10^5.800^
**Male**							
17–21	6	10^6.094^_a_	10^4.746^	10^6.285^	10^5.896^	51.6	10^6.636^
23–27	11	10^6.307^_a_	10^5.634^	10^6.586^	10^6.065^	10^2.260^	10^7.117^
29–33	7	10^6.188^_a_	10^5.472^	10^6.323^	10^5.900^	10^3.418^	10^6.742^
**Female**							
17–21	11	10^4.937^_a_	10^4.526^	10^5.220^	10^4.699^	10^2.215^	10^5.758^
23–27	20	10^6.167^_a_	10^5.498^	10^6.374^	10^5.724^	13.4	10^6.844^
29–33	6	10^5.155^_a_	10^4.512^	10^5.433^	10^5.044^	10^4.187^	10^5.842^

DPI: days post-infestation; *n*: number of observations; $\bar{x}$: arithmetic mean; Md: median; SD: standard deviation; SE: standard error; Min: minimum; Max: maximum. Values followed by the same letter in the same column do not differ significantly by the Kruskal–Wallis test at 5% significance (*p* < 0.05).

**Table 3:** Descriptive analysis of the mean parasitic load values obtained from the qPCR results for the diagnosis of *T. equi* in pooled salivary gland samples and gut samples of *R. (B.) microplus* during the three experimental periods.

DPI	*n*	$\bar{x}$	Md	SD	SE	Min	Max
**Salivary Gland**							
17–21	8	10^3.666^_a_	10^2.928^	10^4.034^	10^3.582^	10^1.223^	10^4.495^
23–27	15	10^5.832^_a_	10^4.657^	10^6.254^	10^5.666^	10^0.995^	10^6.846^
29–33	12	10^4.694^_a_	10^4.515^	10^4.736^	10^4.196^	10^2.833^	10^5.239^
**Gut**							
17–21	7	10^4.593^_a_	10^2.412^	10^4.911^	10^4.489^	10^1.270^	10^5.338^
23–27	13	10^4.205^_a_	10^3.468^	10^4.366^	10^3.809^	10^0.816^	10^4.814^
29–33	11	10^5.142^_a_	10^2.526^	10^5.620^	10^5.099^	10^0.916^	10^6.143^

DPI: days post-infestation; *n*: number of observations; $\bar{x}$: arithmetic mean; Md: median; SD: standard deviation; SE: standard error; Min: minimum; Max: maximum. Values followed by the same letter in the same column do not differ significantly by the Kruskal–Wallis test at 5% significance (*p* < 0.05).
